# Time pressure and deliberation affect moral punishment

**DOI:** 10.1038/s41598-024-67268-3

**Published:** 2024-07-16

**Authors:** Ana Philippsen, Laura Mieth, Axel Buchner, Raoul Bell

**Affiliations:** Department of Experimental Psychology, Heinrich Heine University Düsseldorf, Universitätsstrasse 1, 40225 Düsseldorf, Germany

**Keywords:** Cooperation, Punishment, Cognitive resources, Deliberation, Multinomial processing tree model, Human behaviour, Psychology

## Abstract

The deliberate-morality account implies that moral punishment should be decreased with time pressure and increased with deliberation while the intuitive-morality account predicts the opposite. In three experiments, moral punishment was examined in a simultaneous one-shot Prisoner’s Dilemma game with a costly punishment option. The players cooperated or defected and then decided whether or not to punish their partners. In Experiment 1, the punishment decisions were made without or with time pressure. In Experiment 2, the punishment decisions were immediate or delayed by pauses in which participants deliberated their decisions. In Experiment 3, participants were asked to deliberate self-interest or fairness before deciding whether to punish their partners. Different types of punishment were distinguished using the cooperation-and-punishment model. In Experiment 1, time pressure decreased moral punishment. In Experiment 2, deliberation increased moral punishment. So far, the evidence supports the deliberate-morality account. Experiment 3 demonstrates that the effect of deliberation depends on what is deliberated. When participants deliberated self-interest rather than fairness, moral punishment was decreased. The results suggest that unguided deliberation increases moral punishment, but the effects of deliberation are modulated by the type of deliberation that takes place. These results strengthen a process-based account of punishment which offers a more nuanced understanding of the context-specific effect of deliberation on moral punishment than the deliberate-morality account.

## Introduction

Cooperation forms the foundation of successful societies^1^. While cooperation among kin is a common aspect of animal behavior, humans are special in their capacity to cooperate extensively among non-kin^2^. Humans even cooperate in anonymous one-shot interactions although they cannot expect direct reciprocity^3^. Given that cooperation implies accepting costs to help others, this raises the question: What factors contribute to promoting cooperation in one-shot interactions? One factor that helps to sustain cooperation is the punishment of defection coinciding with one’s own cooperation, referred to as moral punishment^4,5^. Despite its potential costs, people reliably engage in moral punishment even in anonymous one-shot interactions^6^. Since moral punishment plays a critical role in promoting cooperation, it is important to understand what processes underlie this valuable behavior.

Two conflicting positions can be contrasted: The deliberate-morality account^7,8^ implies that people intuitively act selfishly, therefore shying away from the potential costs of moral punishment. This natural tendency to avoid personal costs may stop them from engaging in moral punishment unless their intuitive tendency is overridden by deliberation. From these assumptions, one can derive the hypothesis that moral punishment should be *decreased* under time pressure and increased when deliberation is encouraged and sufficient time is available. In contrast, strong moral norms guide people’s intuitive responses according to the intuitive-morality account^9,10^ which implies that people’s intuition is to morally punish others for refusing to cooperate. Only upon deliberation should they take into account the potential costs of this behavior which then causes them to suppress their natural tendency to morally punish others. From this account, one can derive the hypothesis that moral punishment should be *increased* under time pressure and decreased when deliberation is encouraged and sufficient time is available. In the present experiments, we test these conflicting accounts in a simultaneous one-shot Prisoner’s Dilemma game. We manipulated whether the participants’ decision to punish the partners had to be made under time pressure (Experiment 1) or after a delay in which participants were encouraged to deliberate their decisions (Experiments 2 and 3).

**Figure 1:**
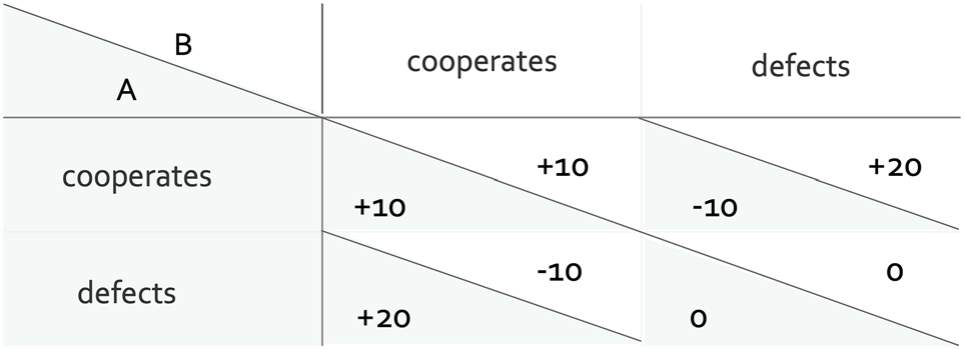
Payoffs in the Prisoner’s Dilemma game as a function of both players’ decisions. Shaded cells mark the decision and payoff of Player A, white cells mark the decision and payoff of Player B.

On a collective level, the best outcome is typically achieved when individuals cooperate with each other. However, when cooperating the individual bears costs. This clash of collective and individual interests is called a social dilemma^11^. To study human decision making in such dilemmas, researchers often rely on economic games in which the complexities of the social dilemma are broken down to a simple payoff matrix. A well-established paradigm for studying cooperation is the Prisoner’s Dilemma^12^. In the Prisoner’s Dilemma, two players simultaneously decide to either cooperate or defect. Depending on their decisions, different outcomes arise, as is illustrated in Fig. 1: For both players, the highest collective outcome is achieved through mutual cooperation. The highest individual outcome, however, is achieved by unilaterally defecting on a cooperating partner who, in turn, receives the worst outcome of the game. For each individual player, there is thus always a financial incentive to defect regardless of what the other player does, while collectively mutual cooperation is better than mutual defection.

Free riders tend to act on the individual rather than the collective interest and exploit others’ cooperation. If too many people free ride, cooperation loses its appeal and declines to alarmingly low levels^13–15^. Here, moral punishment comes into play. The punishment of defectors offers a solution to the free-rider problem as it counters the incentive to defect, thereby effectively enforcing cooperation^16–18^. People readily sacrifice their own resources in social dilemma games to punish others for defection—even in anonymous one-shot interactions where they cannot build a reputation or coerce others into cooperation^19–22^. Due to its crucial role for promoting cooperation, the punishment of unilateral defection is typically interpreted as a behavior that enforces moral norms^16,23^ which is why the punishment of defection by cooperating players is referred to as *moral punishment*^4,5^.

Behaviors such as cooperation, moral punishment or telling the truth are considered prosocial in that they support the collective good at the expense of personal cost. Considerable research has been devoted to the question of whether such behaviors occur deliberately or intuitively [cf. 24]. This distinction between intuitive and deliberate processes lies at the core of dual-processing theories that conceptualize human behavior as an interplay of two different processing types which depend on the cognitive resources available in the situation^25–27^. Type-I processing defines default reactions that arise rapidly and automatically in situations with limited resources. Behaviors based on Type-I-processing are therefore qualified as intuitive reactions. Type-II-processing, in contrast, can overrule intuitive Type-1 processing and guide behavior in a deliberate way, given sufficient time and cognitive resources. To stimulate behavior that relies on intuition rather than deliberation, researchers may impose time pressure or cognitive load. Alternatively, researchers may stimulate behavior that relies on deliberation by requiring decisions to be made after a delay in which participants are encouraged to deliberate their decisions.

Research on how intuition and deliberation affect moral decision-making has yielded mixed results. For instance, there are diverging findings regarding the question of whether intuition or deliberation leads people to tell the truth despite incentives to lie^28–31^. Consistent with this broader literature on moral decision-making, the question of whether moral punishment relies on intuition or deliberation has also produced inconsistent results. This question has as yet been mainly addressed by examining people’s behavior in the Ultimatum Game. In the Ultimatum Game, one player, the proposer, is endowed with a certain amount of money and is asked how much of that money they want to offer to the other player, the responder, and how much they want to keep for themselves. The responder can then decide to accept the offer, leading to the shares being paid out according to the proposer’s offer, or to reject the offer in which case neither player receives any money. As rejecting an offer entails sacrificing own money to ensure that the proposer does not receive an unfair share, rejection in the Ultimatum Game is often interpreted as a form of moral punishment^32–34^. Rejection rates in the Ultimatum Game were found to be increased with restricted cognitive resources^35–38^ and decreased with deliberation during a time delay^39–42^. These findings favor the idea that moral punishment relies on intuition rather than deliberation and thus support the *intuitive-morality account of punishment*. In other studies, however, these results were not replicated^43–48^ or the opposite pattern was found with decreased rejection rates under cognitive load^49,50^ and increased rejection rates with a time delay^51^. These latter results corroborate the *deliberate-morality account of punishment* which is further supported by neuro-imaging studies indicating that the application of punishment relies on areas of cognitive control^52–54^. In sum, the pertinent findings involving the Ultimatum Game are inconsistent. The interpretation of these findings is further complicated by the fact that the Ultimatum Game does not allow to clearly distinguish the participants’ inclination to punish from their inclination to cooperate as both are intertwined in one decision [accept or reject], cf.^5,55^. It is thus interesting to test the effect of time pressure and deliberation on moral punishment in a paradigm that allows to more precisely differentiate between cooperation and punishment.

One such paradigm is the Prisoner’s Dilemma game with costly punishment option^5,56–58^. In the Prisoner’s Dilemma game, both players decide simultaneously whether to cooperate or to defect. Following this decision, they are informed about the outcome of the Prisoner’s Dilemma game. They then decide whether or not to punish their partners by investing some of their own money to deduct money from the partner’s account. To clearly separate the decision processes underlying cooperation and punishment a multinomial processing tree (MPT) model was used to analyze the present data. MPT models are useful tools that serve to disambiguate observable responses by decomposing them into different underlying latent processes^59,60^. Easy-to-read tutorials^61^ and user-friendly software^62^ have facilitated the application of these models in a variety of fields^60^, including moral judgements and decision making^63–70^.

**Figure 2:**
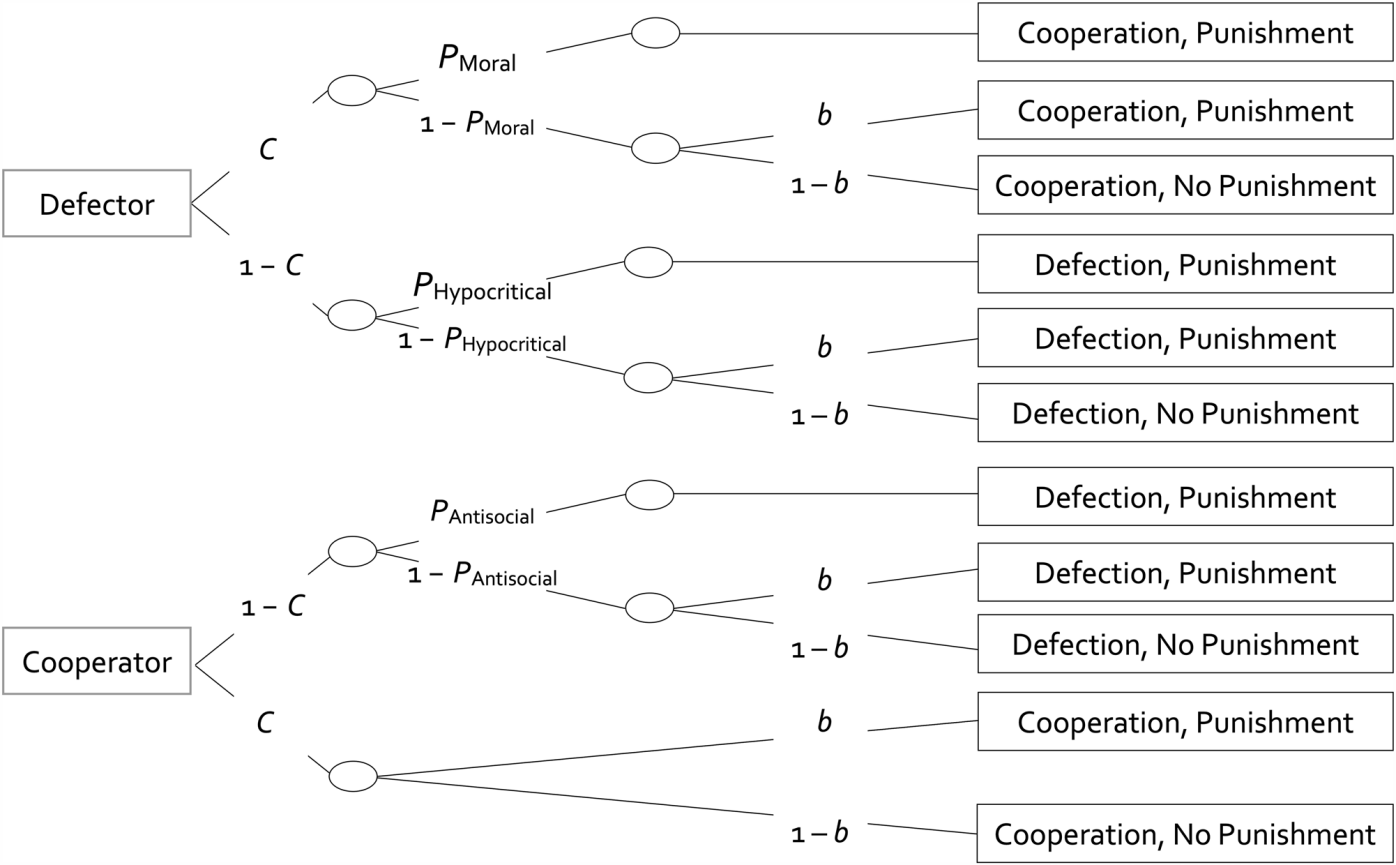
Graphical illustration of the cooperation-and-punishment model. The rectangles on the left represent the two types of partners in the Prisoner’s Dilemma game (defector or cooperator). The rectangles on the right represent the participants’ observable responses (cooperation or defection; punishment or no punishment). The letters along the branches represent the parameters for cooperation (*C*), moral, hypocritical and antisocial punishment (*P*_Moral_, *P*_Hypocritical_ and *P*_Antisocial_, respectively) and the punishment bias (*b*).

The multinomial cooperation-and-punishment model (see Fig. 2) serves to separately measure cooperation, moral punishment, hypocritical punishment, antisocial punishment and a punishment bias^5,56–58^. According to the model, a participant decides to cooperate with probability *C* or to defect with probability 1 − *C*. In a simultaneous Prisoner’s Dilemma game, the partner’s behavior is revealed only after the participant has made their decision to cooperate or to defect. When the participant decides whether they want to cooperate or to defect, the participant thus cannot know whether they interact with a defecting or cooperating partner. The model therefore implies that parameter *C* does not differ as a function of the behavior of the partner. The *P*_•_ parameters refer to the conditional probabilities of different types of punishment that are specifically elicited by, and thereby contingent upon, the outcomes of the Prisoner’s Dilemma game. These can be contrasted with an unspecific punishment bias *b* that is assumed to be unaffected by the outcome of the Prisoner’s Dilemma game. To illustrate, if a cooperating participant interacts with a defecting partner, the participant may apply *moral punishment* with the conditional probability *P*_Moral_. If moral punishment is not applied which occurs with the conditional probability 1 − *P*_Moral_, the participant may still punish the partner due to an unspecific punishment bias with the conditional probability *b*. With the conditional probability 1−*b*, no punishment is applied. If a defecting participant interacts with a defecting partner, the participant may apply *hypocritical punishment* with the conditional probability *P*_Hypocritical_. This type of punishment can be considered hypocritical as it enforces a norm of cooperation the participant themselves failed to follow. If no hypocritical punishment is applied which occurs with the conditional probability 1 − *P*_Hypocritical_, the participant may still punish the partner due to the unspecific punishment bias with the conditional probability *b*. With the conditional probability 1 − *b*, no punishment is applied. If a defecting participant interacts with a cooperating partner (lower tree of Fig. 2), the participant may apply *antisocial punishment* with the conditional probability *P*_Antisocial_. This type of punishment is termed antisocial because it directly opposes the cooperation norm. If no antisocial punishment is applied which occurs with the conditional probability 1 − *P*_Antisocial_, the participant may still punish the partner due to the unspecific punishment bias with the conditional probability *b*. With the conditional probability 1 − *b*, no punishment is applied. Mutual cooperation does not provide any specific reason to punish the partner. The punishment that still occurs in this condition is thus assumed to be caused only by the punishment bias *b*, representing an unspecific tendency to punish the partner irrespective of the outcome of the Prisoner’s Dilemma game^5,56–58^. For example, cognitive load has been demonstrated to increase participants’ inclination to indiscriminately punish partners in the Prisoner’s Dilemma game^5^, highlighting the necessity of accounting for bias when analyzing the punishment data, especially when manipulating the availability of cognitive resources. The concept of the punishment bias *b* is parallel to how response bias is taken into account in other multinomial decision-making models^71–75^.

Here, a note of caution is in order: While we use the adjectives “moral,” “hypocritical,” and “antisocial” to refer to the punishment parameters that are clearly defined in the cooperation-and-punishment model, these labels should not be overinterpreted, particularly not against the backdrop of how these adjectives are used in everyday language. For instance, “moral” punishment may sometimes be influenced by self-interested motivations such as seeking retribution or reputation building. Furthermore, in everyday language “hypocritical” punishment may also encompass moral or antisocial motivations. Here, these adjectives merely serve as easily accessible descriptors for the parameters to simplify communication across disciplines and are not intended as exhaustive definitions of the parameters. The strength of the cooperation-and-punishment model lies in its precise structural definitions of parameters, as illustrated in Fig. 2, which remain valid regardless of the specific adjectives used as parameter labels. Therefore, the applicability of the model is not dependent on the adjectives used as verbal labels of the parameters.

The validity of the cooperation-and-punishment model has been demonstrated in various studies in which the model has been successfully used to separately measure cooperation, the different types of punishment and the punishment bias^5,56–58^. In one of these studies, Mieth et al.^5^ have restricted participants’ cognitive resources in the Prisoner’s Dilemma game using a concurrent distractor task. This induction of cognitive load decreased moral punishment compared to a control group without distraction. The effect was specific to moral punishment. Hypocritical and antisocial punishment remained unaffected by cognitive load. The unspecific punishment bias was increased under cognitive load, suggesting that punishment was applied less purposefully when distracted by another task. These findings show that an increased availability of cognitive resources causes participants to apply moral punishment to enforce norms of cooperation, in support of the general idea that the moral use of punishment is facilitated by deliberation. This deliberate-morality interpretation implies that time pressure, like cognitive load, should specifically decrease moral punishment. Conversely, a delay during which participants are encouraged to deliberate their decisions should have the opposite effect, thereby increasing moral punishment. However, manipulations of cognitive load and time pressure have not always produced convergent results^24^. The aim of the present series of experiments was thus to dissect the effects of time pressure and deliberation on costly punishment in the Prisoner’s Dilemma game. If deliberation causes punishment to be applied in a purposeful moral fashion, moral punishment should be decreased with time pressure (Experiment 1) and increased when deliberation is encouraged (Experiment 2). Furthermore, the deliberation manipulation is extended in the final experiment in which we tested whether the effect of deliberation depends on *what* is deliberated (Experiment 3): One group of participants was encouraged to deliberate self-interest while the other group was encouraged to deliberate fairness. This final experiment was performed to challenge the dichotomy that lies at the core of the dual-processes models. Specifically, the experiment served to test whether fairness-focused deliberation would favor moral punishment relative to self-interest-focused deliberation, consistent with a more nuanced process-based account according to which the effect of deliberation depends on the specific processes involved^52,76^.

## Experiment 1

Imposing a time constraint is a classical method to manipulate the availability of cognitive resources^10,77,78^. In line with this established approach for suppressing deliberation^24^, participants had to make punishment decisions either without or with time pressure. Following the deliberate-morality account of punishment and the assumption that time pressure suppresses deliberation^7^, moral punishment should be decreased in the condition with time pressure relative to the condition without time pressure. By contrast, the intuitive-morality account of punishment^9^ implies moral punishment to be increased by time pressure.

### Method

#### Participants and design

A total of 217 participants took part in the online study that was conducted via the online platform SoSci Survey^79^. Participants were recruited via a mailing list to which people could subscribe if they wished to participate in psychology experiments and with the help of social media channels directed at Heinrich Heine University students. Data had to be excluded from analyses for the following reasons: The data were not stored properly (*n* = 6), the participant was younger than 18 and thus could not legally consent to participate (*n* = 1), the participant stated to have poor eyesight (*n* = 1) or the participant withdrew their consent to the use of their data at the end of the study (*n* = 3). The final sample consisted of *N* = 206 participants (154 female, 51 male, 1 non-binary) who were between 18 and 66 (*mean age* = 25, *standard deviation* = 10) years old. Of these, 102 were assigned to the condition without time pressure and 104 participants were assigned to the condition with time pressure. The median duration of participation was 10 min. Undergraduate psychology students could receive course credit for their participation. Other students or non-students had the chance to win a shopping voucher worth 20 €; these participants knew that only one voucher was available which would be awarded through lottery after data collection had been completed. A sensitivity analysis with G*Power^80^ showed that, with α = 0.05, *N* = 206 participants and 20 behavioral choices in the Prisoner’s Dilemma game, effects of time pressure on the different types of punishment as small as *w* = 0.06 could be detected with a statistical power of 1 − β = 0.95.

#### Ethics

The present series of experiments was approved by the Ethics Committee of the Faculty of Mathematics and Natural Sciences of the Heinrich-Heine-University Düsseldorf and conducted in accordance with the requirements of the Declaration of Helsinki. All participants were informed that they would play a game involving a number of interactions with partners who would simultaneously make the same decision as themselves and that the purpose of the study was to gain insight into people’s behavior in interactions. They then gave written informed consent before participating in the experiment. At the end of each experiment, participants were debriefed that the purpose of the experiment was to study how decision time affects cooperation and punishment. They were informed that they had interacted with programmed partners during the experiment and were then reminded that they could still withdraw their consent to the use of their data.

#### Prisoner’s Dilemma

Participants played the Prisoner’s Dilemma game with a costly punishment option which has been used in several previous studies to study cooperation and punishment^5,56–58,81,82^. At the start of the Prisoner’s Dilemma game, participants were endowed with 400 cents (4 €). They were informed that they would receive an online-shopping voucher equivalent to the amount of money they had in their account balance at the end of the experiment (347 cents on average, *standard deviation* = 55). Each participant played 20 rounds of the Prisoner’s Dilemma game with a costly punishment option with 20 different partners. Half of the partners cooperated and the other half defected. Each participant saw the partners in a different, randomly determined order.

**Figure 3:**
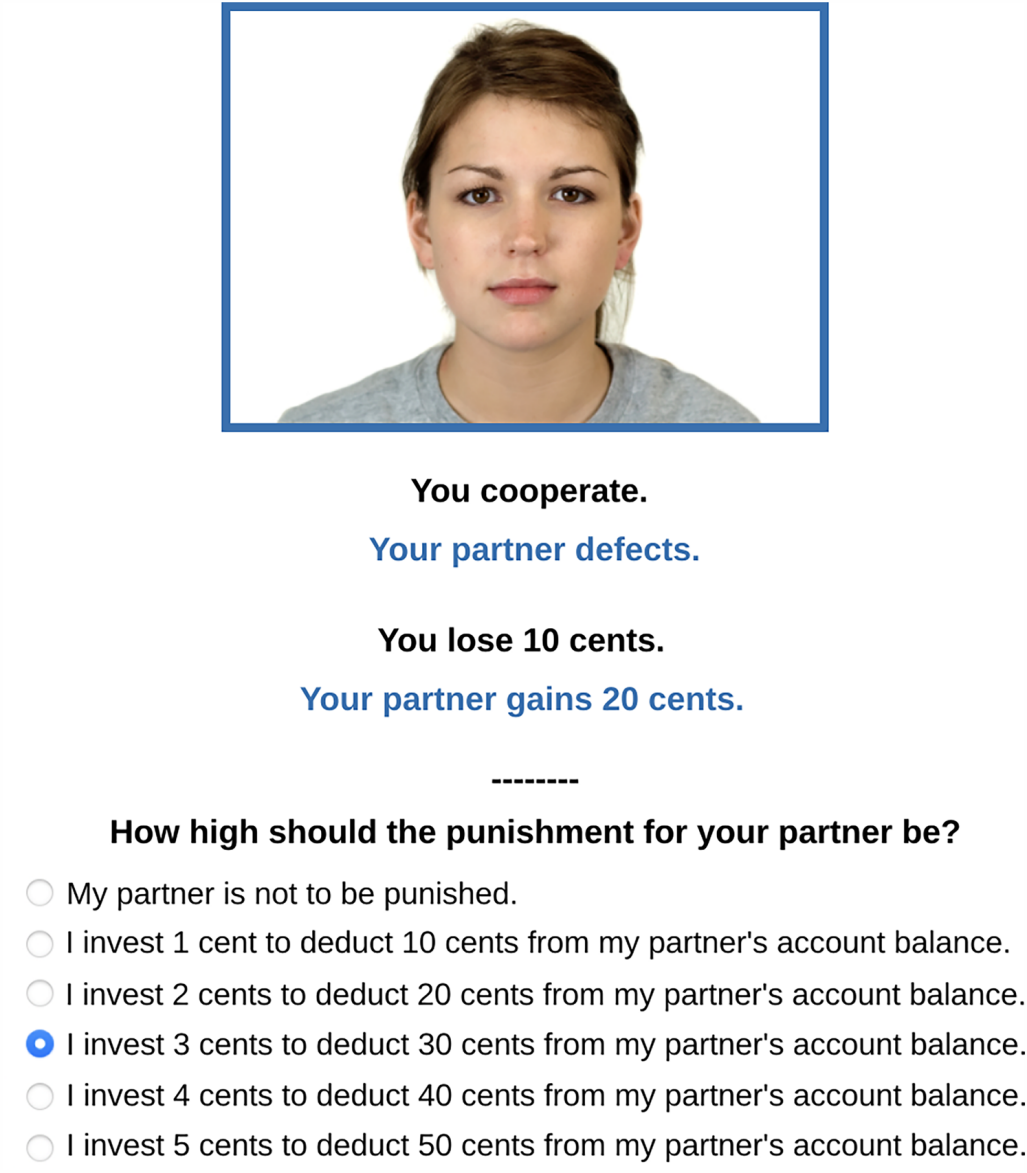
Example trial of the Prisoner’s Dilemma with punishment option. In this example trial, the participant cooperated and the partner defected. Therefore, the participant lost 10 cents and the partner gained 20 cents. The participant then decided to punish their partner by investing 3 cents to deduct 30 cents from the partner’s account balance. The facial photograph of the partner was randomly selected from a set of 10 male and 10 female faces taken from the Chicago Face Database^83^. Informed consent to publish the figure in an online open-access publication has been obtained.

Before each trial started, the participant was informed about their current account balance. The participant started the trial by clicking a “Continue” button. To emphasize the social nature of the game, the participant saw a facial photograph of their interaction partner. The picture was randomly drawn from 10 male and 10 female faces (between 18 and 40 years old) of the Chicago Face Database^83^. The face of the partner was shown from a frontal view with a neutral expression. The picture had a resolution of 266 × 186 pixels. The participant was asked “Do you want to cooperate or defect?” and answered by selecting either “I cooperate” or “I defect”. The participant was then presented with a summary of the interaction. The participant had previously been instructed that the partner made the decision on whether to cooperate or to defect at the same time as the participant. The participant received feedback about their own decision (e.g., “You cooperate.”) and the partner’s decision (e.g., “Your partner defects”), and how these decisions affected the participant’s account balance (e.g., “You lose 10 cents.”) and the partner’s account balance (e.g., “Your partner gains 20 cents.”). The feedback regarding the participant’s decision and outcome was displayed in black, while the feedback regarding the partner’s decision and outcome was displayed in blue, corresponding to the blue frame surrounding the partner’s photograph (see Fig. 3). The face of the interaction partner and the feedback remained visible until after the end of each trial.

Mutual cooperation resulted in a moderate gain (+ 10 cents) for both partners. Mutual defection resulted in neither a gain nor a loss (0 cents) for each partner. In the case of unilateral cooperation, the defecting partner made a large profit (+ 20 cents) at the expense of the cooperating partner who lost money (− 10 cents). The payoff structure thus corresponds to that of a typical Prisoner’s Dilemma game in that there was a high temptation for unilateral defection, a moderate reward for mutual cooperation, no reward after mutual defection and a loss when cooperating with a defecting partner^11^.

#### Costly punishment

Immediately after the interaction in the Prisoner’s Dilemma game, the participant had the option to punish the partner. The participant could decide either not to punish their partner or to invest 1, 2, 3, 4 or 5 cents to deduct 10, 20, 30, 40 or 50 cents, respectively, from their partner’s account balance, as depicted in Fig. 3. They were then automatically forwarded to the next screen which displayed the participant’s investment in punishment in black, the resulting punishment for their partner in blue as well as the partner’s investment in punishment in blue and the resulting punishment for the participant in black. The partners were programmed to morally punish unilateral defection of the participants with a random amount of 10, 20, 30, 40 or 50 cents. This mimics the behavior of real participants who primarily use the punishment option to punish unilateral defection^6,19–21^. Participants then initiated the next round of the game by clicking a “Continue” button.

#### Time-pressure manipulation

Participants were randomly assigned either to the condition without time pressure or to the condition with time pressure. In the condition without time pressure, the participant was encouraged in the instructions and before each interaction to take their time and to deliberate carefully how they wanted to respond. The participant had unlimited time both when deciding whether to cooperate and when deciding whether to punish the partner. In the condition without time pressure, the median response time was 3.6 s for the cooperation decision and 3.5 s for the punishment decision.

In the condition with time pressure, the participant was informed in the instructions and before each interaction to decide quickly whether to cooperate and whether to punish because there would be a time limit of five seconds within which either response had to be made. A countdown from 5 to 0 s was presented until each of these responses had to be made or until the countdown reached 0 s. When participants did not respond within the five seconds, a warning was displayed, asking the participant to respond more quickly. Participants then had to click on a “Continue” button to repeat the trial. In the Prisoner’s Dilemma game, 92% of the 104 participants in the condition with time pressure never exceeded the time limit and another 6% exceeded the time limit only once. The summary of the Prisoner’s Dilemma game interaction was presented with a countdown of five seconds and participants were automatically forwarded to the punishment option when they did not click the “Continue” button within the time limit. When making the punishment decision, 84% of the 104 participants never exceeded the time limit and another 13% exceeded the time limit only once. In the condition with time pressure, the median response time was 2.4 s for the cooperation and 2.4 s for the punishment decision.

## Results

The first four trials had originally been designed as practice trials. Upon a reviewer’s suggestion, these practice trials are now included in the analyses. Whether or not the practice trials are included has no effect on the statistical conclusions in any experiment reported here except that the bias parameter *b* differs between the two conditions in Experiment 3 when the practice trials are included.

**Figure 4:**
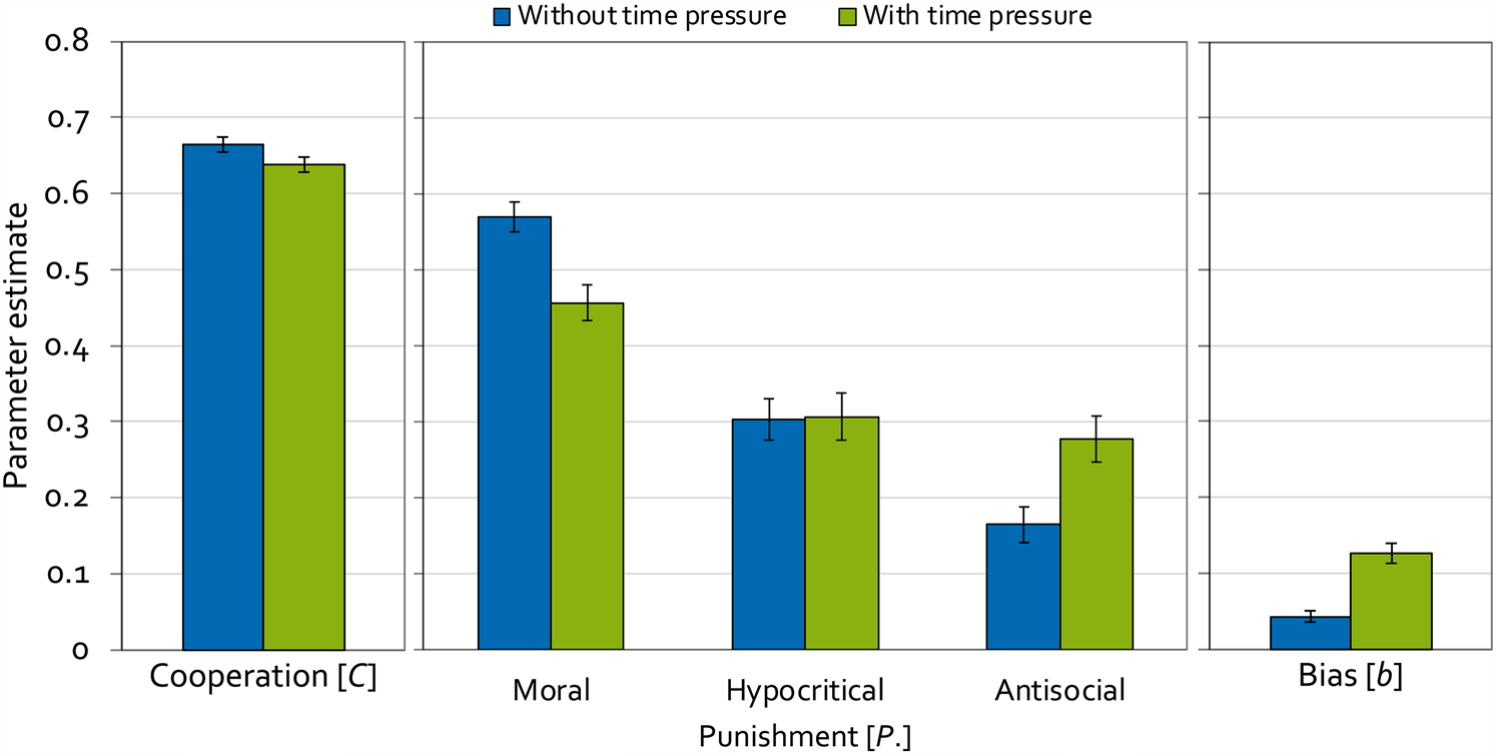
Estimates of the parameters of the cooperation-and-punishment model depending on whether decisions were made without or with time pressure in Experiment 1. Parameter *C* represents the probability of cooperation. Parameters *P*_Moral_, *P*_Hypocritical_ and *P*_Antisocial_ represent the conditional probabilities of moral punishment, hypocritical punishment and antisocial punishment, respectively. Parameter *b* represents the punishment bias, that is, the probability of punishment irrespective of the outcome of the Prisoner’s Dilemma game. The error bars represent the standard errors.

For the model-based analyses, the α level was set to 0.05. Parameter estimates and goodness-of-fit tests were obtained using *multitree*^62^. To analyze the present data, two instances of the model depicted in Fig. 2 are needed, one for the condition without time pressure and one for the condition with time pressure. The base model fit the data, *G*^2 ^(2) = 0.18, *p* = 0.913, indicating that the base model’s parameters reflect the observed data adequately. Figure 4 displays the estimates of the cooperation parameter (left panel), the punishment parameters (middle panel) and the punishment bias (right panel).

Multinomial models make it possible to test hypotheses directly at the level of the model parameters, that is, at the level of the cognitive processes measured by the model. For example, the hypothesis that cooperation is more likely in the condition without time pressure than in the condition with time pressure can be tested by restricting parameter *C* to be equal in the two conditions. If the restricted model fits the data significantly worse than the base model, indicated by the Δ*G*^2^ statistic that is chi-square distributed with degrees of freedom given in parentheses, then the null hypothesis that parameter *C* does not differ between the two conditions needs to be rejected. As a consequence, it can be concluded that cooperation differs between the two conditions^60^. In fact, the cooperation parameter could be equated across conditions without causing a significant decrease in model fit, Δ*G*^2 ^(1) = 3.24, *p* = 0.072, *w* = 0.03. It thus needs to be concluded that cooperation was unaffected by time pressure.

The central hypothesis test in Experiment 1 concerns the parameter representing the probability of moral punishment. In line with the deliberate-morality account of punishment, moral punishment was significantly less likely in the condition with time pressure than in the condition without time pressure, Δ*G*^2 ^(1) = 13.61, *p* < 0.001, *w* = 0.06. As a secondary finding, the probability of hypocritical punishment did not differ as a function of time pressure, Δ*G*^2 ^(1) = 0.01, *p* = 0.936, *w* < 0.01. Antisocial punishment was more likely in the condition with time pressure compared to the condition without time pressure, Δ*G*^2 ^(1) = 8.37, *p* = 0.004, *w* = 0.05. Furthermore, time pressure led to an increase in the punishment bias, Δ*G*^2 ^(1) = 31.40, *p* < 0.001, *w* = 0.09, compared to the condition without time pressure.

## Discussion

Experiment 1 served to examine the effects of time pressure on moral punishment. Moral punishment was significantly decreased when punishment decisions were made with time pressure compared to when they were made without time pressure. This finding supports the deliberate-morality account of punishment according to which time pressure interferes with the moral use of punishment with the social goal of promoting cooperation^7^.

The model-based analysis shows that the suppressive effect of time pressure on punishment was specific to moral punishment. Hypocritical punishment remained unaffected by time pressure. Antisocial punishment increased with time pressure. Furthermore, time pressure significantly increased participants’ bias to indiscriminately punish irrespective of the outcome of the Prisoner’s Dilemma interaction, in line with the prediction that time pressure causes punishment to be applied less purposefully^5^.

It seems noteworthy that the effects of time pressure on punishment observed here are strikingly parallel to the effects of cognitive load on punishment reported by Mieth et al.^5^. Specifically, Mieth et al. have observed that cognitive load decreases moral punishment but has no effect on hypocritical punishment. Antisocial punishment was descriptively, but not significantly, increased in the condition with cognitive load compared to the condition without cognitive load. Furthermore, cognitive load induced an increase in the punishment bias, supporting the idea that the reduced availability of cognitive resources causes punishment to be applied less purposefully. Together with the previous findings^5^, the present findings suggest that manipulations suppressing deliberation have consistent effects on punishment, regardless of whether the suppression of deliberation is caused by cognitive load or time pressure.

## Experiment 2

In Experiment 2, the goal was to extend the empirical basis of the previous findings by testing whether encouraging deliberation has effects on moral punishment opposite to those of suppressing deliberation. The deliberate-morality view of punishment^7^ leads to the prediction not only that moral punishment should become less likely with time pressure but also that moral punishment should become more likely with the time spent deliberating the punishment decision. To test this prediction, punishment decisions were delayed and participants were encouraged to deliberate their punishment decision during the delay in Experiment 2. This condition with deliberation was contrasted to a condition without deliberation in which punishment was not delayed and participants received no instructions encouraging them to deliberate their punishment decision.

### Method

#### Participants and design

A total of 646 participants took part in the online study that was conducted via the platform SoSci Survey^79^. Participants were recruited by the panel provider *mingle* (https://mingle.respondi.de). Data had to be excluded from analyses for the following reasons: The data were not stored properly (*n* = 9), the participant took part repeatedly (*n* = 47), the participant dropped out prematurely (*n* = 78) or the participant withdrew their consent to the use of their data at the end of the study (*n* = 4). The final sample consisted of *N* = 508 participants (189 female, 318 male, 1 non-binary) who were between 19 and 69 (*mean age* = 40, *standard deviation* = 12) years old. Of these, 258 participants were assigned to the condition without deliberation and 250 were assigned to the condition with deliberation. The median duration of participation was 16 min. Participants received their final account balance achieved in the game in addition to their usual compensation by the panel provider (see explanation below). A sensitivity analysis with G*Power^80^ showed that, with α = 0.05, *N* = 508 participants and 20 behavioral choices in the Prisoner’s Dilemma game, effects of deliberation on punishment as small as *w* = 0.04 could be detected with a statistical power of 1 − β = 0.95.

#### Materials and procedure

The materials and procedure were the same as those of Experiment 1 with the following exceptions. Participants of the panel provider *mingle* are used to being compensated with points that can be exchanged for online vouchers, charity donations or money. Participants were therefore informed in the experiment-specific instructions that they played for points (with 1 point corresponding to 1 Euro cent) that they would receive by *mingle* in addition to the points which they already knew they would receive as a compensation for participating from the invitation e-mail they had received by mingle prior to participating. To align their starting endowment with *mingle’s* common compensation fees, participants were endowed with 80 points at the start of the game. The costly punishment option was adjusted to the lower starting endowment to avoid negative account balances. In each trial, participants could thus invest up to three points to deduct a maximum of 30 points from their partner’s account balance. On average, participants achieved a final account balance of 63 points (*standard deviation* = 29).

#### Manipulation of deliberation

Participants were randomly assigned to the condition without deliberation or to the condition with deliberation. In the condition without deliberation, the participant decided (self-paced) whether to punish the partner right after the Prisoner’s Dilemma interaction had been completed. The median response time for the cooperation decision in this condition was 2.9 s and the median response time for the punishment decision was 2.8 s.

In the condition with deliberation, the cooperation decision was made without a delay. The median response time for the cooperation decision was 3.0 s. By contrast, the punishment decision was delayed by 30 s. The participant was instructed beforehand that, following the decision in the Prisoner’s Dilemma game, the participant would be given time to deliberate the punishment decision. The participant was instructed: “Please take your time to carefully deliberate on what you want to do next.” Right after each cooperation decision in the Prisoner’s Dilemma game, the punishment option was presented, but it was accompanied by the instruction to deliberate on whether, and if so, how to punish the partner. The punishment option was initially deactivated. Beneath the punishment option, a countdown from 30 to 0 s was presented. After 30 s, the instruction to deliberate the punishment decision was replaced by the question “How high should the punishment for your partner be?” and the punishment option was activated so that the participant could implement their punishment decision. Including the 30-s delay, the median response time for the punishment decision in the condition with deliberation was 37.4 s.

### Results

**Figure 5:**
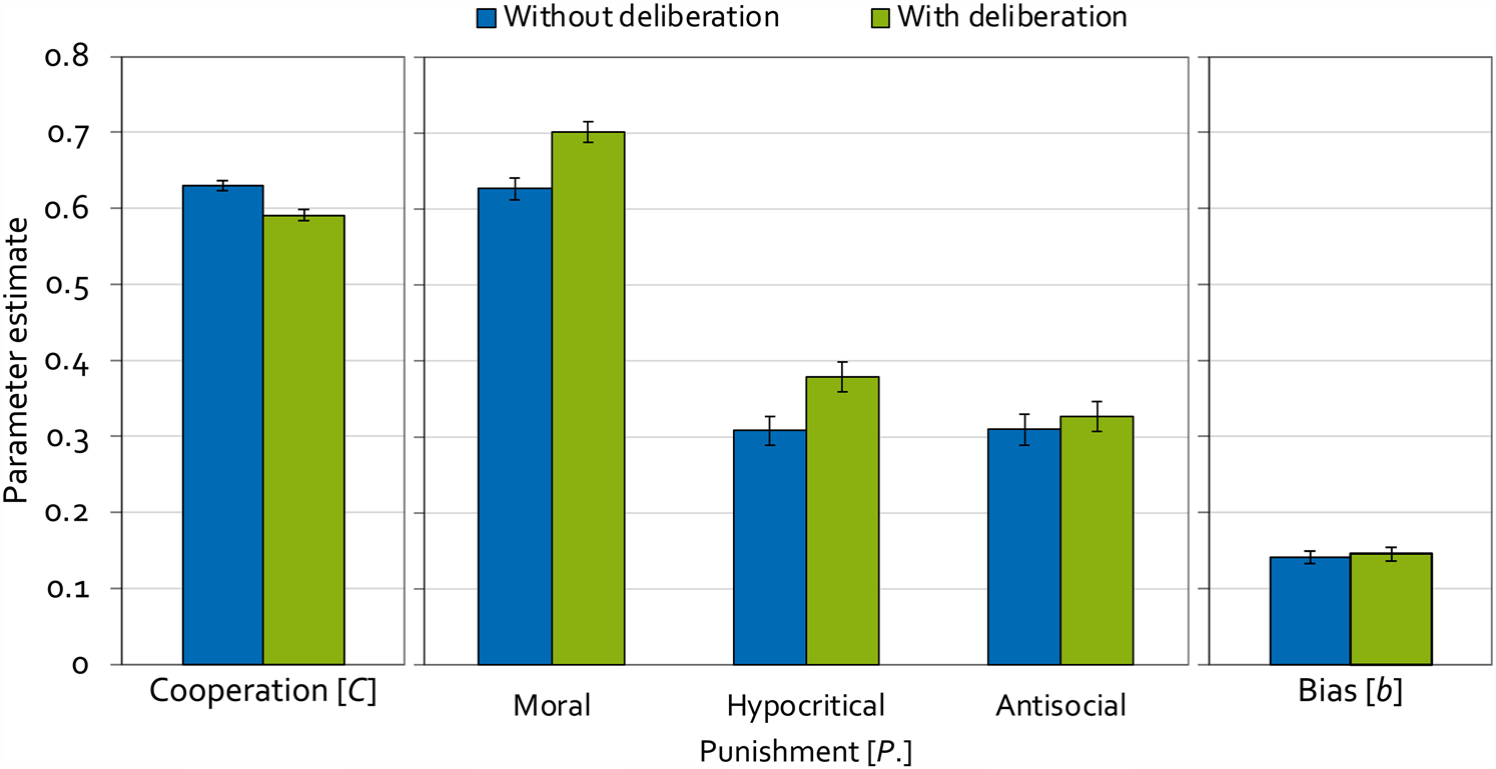
Estimates of the parameters of the cooperation-and-punishment model as a function of condition (without deliberation, with deliberation) in Experiment 2. Parameter *C* represents the probability of cooperation. Parameters *P*_Moral_, *P*_Hypocritical_ and *P*_Antisocial_ represent the conditional probabilities of moral punishment, hypocritical punishment and antisocial punishment, respectively. Parameter *b* represents the punishment bias, that is, the probability of punishment irrespective of the outcome of the Prisoner’s Dilemma game. The error bars represent the standard errors.

To analyze the present results, two instances of the model depicted in Fig. 2 are needed, one for the condition without deliberation and one for the condition with deliberation. The base model fit the data, *G*^2 ^(2) = 1.67, *p* = 0.434, indicating that the base model’s parameters reflect the observed data adequately. Figure 5 displays the estimates of the cooperation parameter (left panel), the punishment parameters (middle panel) and the punishment bias (right panel). The probability to cooperate was significantly lower in the condition with deliberation compared to the condition without deliberation, Δ*G*^2 ^(1) = 16.28, *p* < 0.001, *w* = 0.04.

The central hypothesis test again concerns the parameter representing the probability of moral punishment. In line with the deliberate-morality account of punishment, moral punishment was significantly more likely in the condition with deliberation compared to the condition without deliberation, Δ*G*^2 ^(1) = 14.64, *p* < 0.001, *w* = 0.04. As a secondary finding, hypocritical punishment was also more likely in the condition with deliberation compared to the condition without deliberation, Δ*G*^2 ^(1) = 6.45, *p* = 0.011, *w* = 0.03. Neither antisocial punishment, Δ*G*^2 ^(1) = 0.40, *p* = 0.529, *w* = 0.01, nor the punishment bias, Δ*G*^2 ^(1) = 0.14, *p* = 0.711, *w* < 0.01, were affected by the deliberation manipulation.

### Discussion

The aim of Experiment 2 was to extend the results of the previous experiment which demonstrated that time pressure decreased moral punishment. From the deliberate-morality account, the prediction can be derived that a time delay during which one is encouraged to deliberate one’s punishment decision should have an effect on moral punishment that is opposite to the effect of time pressure. In line with this prediction, deliberation significantly increased moral punishment compared to a condition without deliberation, providing further support for the idea that deliberation favors the moral use of punishment.

Whereas the time-pressure manipulation of Experiment 1 did not affect hypocritical punishment, deliberating one’s punishment decision in Experiment 2 increased hypocritical punishment relative to the condition without deliberation. Furthermore, deliberation had no effect on antisocial punishment or the punishment bias. These findings thus suggest that the lack of resources, caused by cognitive load and time pressure, affects decision-making in ways that are not simply the mirror image of deliberation.

While the decrease of cooperation in the condition with deliberation in comparison to the condition without deliberation at first glance seems to support an intuitive-morality view on cooperation^10^, it is important to note that the manipulation of deliberation in Experiment 2 consisted primarily of delaying the punishment decisions. Decisions to cooperate or defect were not delayed. While the manipulation also included an instruction to “please take your time to carefully deliberate on what you want to do next”, this instruction referred explicitly to the punishment and not to the cooperation decision. However, it cannot be ruled out that these instructions as well as deliberating about the punishment decisions may have caused participants to generally adopt a more deliberate processing mode, explaining why the manipulation also affected their propensity to cooperate. Note that this is only a post-hoc interpretation that requires confirmation in future studies before firm conclusions can be drawn about this issue.

## Experiment 3

In the previous two experiments, we found evidence in favor of a deliberate-morality account of punishment. While this is in line with the results of some studies^49,50^, it is in opposition to others that suggest punishment is intuitive rather than deliberate^35–37^. Such inconsistencies have motivated Declerck and Boone^52^ to move away from the strict dichotomy of intuitive and deliberate moral behaviors. Instead, they proposed a process-based account according to which intuition and deliberation can either favor or inhibit moral behaviors, depending on contextual factors. For instance, according to this account, unguided deliberation in Experiment 2 may have increased moral punishment because participants were more likely to deliberate fairness than to deliberate self-interest. However, this does not mean that deliberation will always favor moral behaviors as the effect of deliberation may depend on *what* is deliberated which, in turn, may depend on the general context in which deliberation takes place. If participants are encouraged to deliberate moral concerns, this should have an enhancing effect on moral punishment. If participants are encouraged to deliberate self-interest, this should have a diminishing effect on moral punishment. In Experiment 3, this hypothesis was put to an empirical test. The punishment decision was delayed by a pause in which participants were explicitly encouraged to deliberate either fairness or self-interest, depending on the experimental condition they were assigned to. Instructions to deliberate fairness should increase moral punishment relative to instructions to deliberate self-interest.

### Method

#### Participants and design

A total of 698 participants took part in the online study that was conducted via the platform SoSci Survey^79^. As in Experiment 2, participants were recruited by the panel provider *mingle*. Data had to be excluded from analyses for the following reasons: The participant took part repeatedly (*n* = 45), the participant dropped out prematurely (*n* = 124) or the participant did not consent to the use of their data at the end of the study (*n* = 2). The final sample consisted of *N* = 527 participants (206 female, 320 male, 1 non-binary) who were between 18 and 70 (*mean age* = 45, *standard deviation* = 14) years old. Of these, 255 participants were assigned to the self-interest-deliberation condition and 272 were assigned to the fairness-deliberation condition. The median duration of participation was 22 min. As in Experiment 2, participants received their final account balance in the game in addition to their usual compensation by the panel provider. A sensitivity analysis with G*Power^80^ showed that, with α = 0.05, *N* = 527 participants and 20 behavioral choices in the Prisoner’s Dilemma game, effects of deliberation on punishment as small as *w* = 0.04 could be detected with a statistical power of 1 − β = 0.95.

#### Materials, procedure and manipulation

The materials were the same as those of Experiments 1 and 2. The experimental procedure in Experiment 3 corresponded to the condition with deliberation of Experiment 2. The cooperation decisions were not delayed. The median response times for the cooperation decision were 4.5 s in the self-interest-deliberation condition and 4.7 s in the fairness-deliberation condition. By contrast, the punishment decision was delayed by a 30-s pause in which the participant was asked to deliberate the punishment decision. In Experiment 3, however, the specific content on which to deliberate was manipulated. In the self-interest-deliberation condition, the participant was instructed to deliberate “whether it is profitable to punish your partner and what a punishment means for your own account balance”. The median response time for the punishment in the self-interest-deliberation condition was 38.0 s. In the fairness-deliberation condition, the participant was instructed to deliberate “how fair or unfair your partner’s behavior was and whether your partner deserves a punishment for this behavior”. The median response time for the punishment in the fairness-deliberation condition was 38.4 s. The same instruction was also included in the written instructions provided before the start of the Prisoner’s Dilemma game. The remaining instructions and procedure were identical to those of Experiment 2 with the exception that the starting endowment was increased to 100 points (corresponding to 100 cents or 1 €). On average, participants achieved a final account balance of 85 points (*standard deviation* = 36).

### Results

**Figure 6:**
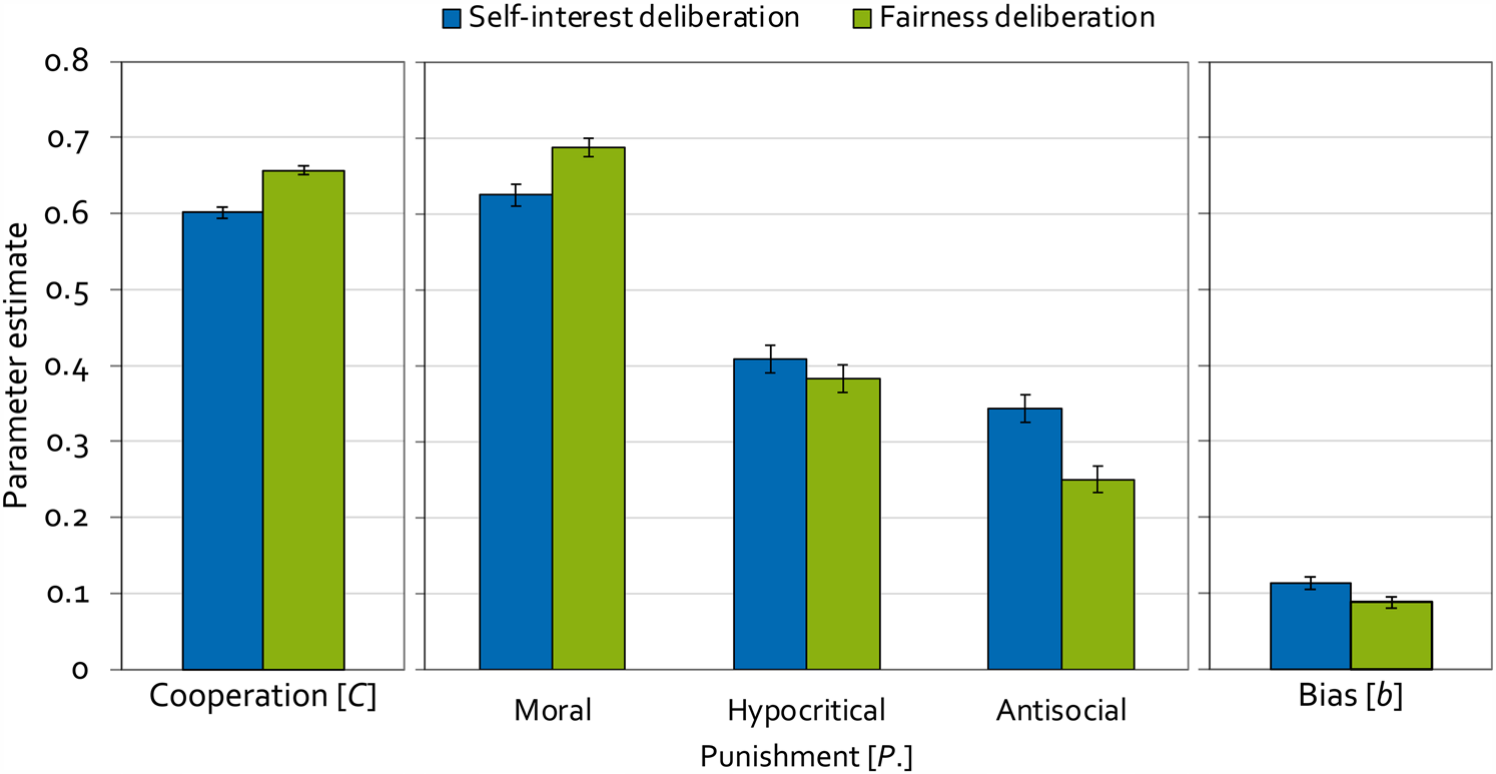
Estimates of the parameters of the cooperation-and-punishment model as a function of the content of deliberation (self-interest, fairness) in Experiment 3. Parameter *C* represents the probability of cooperation. Parameters *P*_Moral_, *P*_Hypocritical_ and *P*_Antisocial_ represent the conditional probabilities of moral punishment, hypocritical punishment and antisocial punishment, respectively. Parameter *b* represents the punishment bias, that is, the probability of punishment irrespective of the outcome of the Prisoner’s Dilemma game. The error bars represent the standard errors.

To analyze the present results, two instances of the model depicted in Fig. 2 are needed, one for the self-interest-deliberation condition and one for the fairness-deliberation condition. The base model fit the data, *G*^2 ^(2) = 0.36, *p* = 0.837, indicating that the base model’s parameters reflect the observed data adequately. Figure 6 displays the estimates of the cooperation parameter (left panel), the punishment parameters (middle panel) and the punishment bias (right panel). The probability to cooperate was significantly lower when participants deliberated self-interest compared to when they deliberated fairness, Δ*G*^2^ (1) = 35.17, *p* < 0.001, *w* = 0.06.

The central hypothesis test again concerns the parameter representing the probability of moral punishment. In line with the process-based account according to which the effect of deliberation on moral punishment depends on the content of deliberation, moral punishment was significantly less likely when participants deliberated self-interest than when they deliberated fairness, Δ*G*^2 ^(1) = 11.63, *p* < 0.001, *w* = 0.03. As a secondary finding, hypocritical punishment did not differ as a function of the content of deliberation, Δ*G*^2 ^(1) = 0.96, *p* = 0.327, *w* = 0.01. Antisocial punishment was more likely when participants deliberated self-interest compared to when they deliberated fairness, Δ*G*^2 ^(1) = 13.38, *p* < *0.0*01, *w* = 0.04. Further, the punishment bias was significantly higher when participants deliberated self-interest than when they deliberated fairness, Δ*G*^2 ^(1) = 5.67, *p* = 0.017, *w* = 0.02.

#### Discussion

The aim of Experiment 3 was to test whether moral punishment depends on the content of deliberation. The experimental procedure was identical to the condition with deliberation used in Experiment 2, but participants were instructed to deliberate either self-interest or fairness. Moral punishment was more likely when participants were instructed to deliberate fairness rather than self-interest. These results demonstrate that the effect of deliberation on moral punishment depends on contextual factors such as, in the present case, the content of deliberation. This finding supports the process-based account of punishment^52^ implying that, to fully understand the effects of deliberation on moral behaviors, it is necessary to move away from the strict dichotomy that lies at the core of dual-process theories. Previous studies have already hinted at contextual factors that might modulate the effect of deliberation on punishment^37,46,51,84–86^. Here we extend these findings by demonstrating that the instructed content of deliberation affects moral punishment.

Even when participants deliberated their self-interest, they still morally punished unilateral defection with a high probability although this entailed sacrificing own costs for no apparent benefit and thereby went against their self-interest. Previous studies using the cooperation-and-punishment model have already demonstrated the robustness of participants’ inclination to morally punish unilateral defection which remained at a high level even when, for example, the majority of partners defected^58^ or when participants were offered other ways to communicate their anger in response to the defection^57^. Taken together, these findings suggest that, despite being sensitive to contextual factors, people have a strong and robust preference for moral punishment which aligns with the moral preference hypothesis proposed by Capraro and Perc^87^.

In contrast to moral punishment, antisocial punishment was more likely when participants deliberated their self-interest compared to when they deliberated fairness. This underlines the idea that antisocial punishment is antithetical to the very fairness norms that moral punishment is assumed to promote^88,89^. Specifically, it has been suggested that antisocial punishment serves to directly oppose the normative pressure towards cooperation^58^, implying that individuals are more likely to engage in antisocial punishment when they prioritize self-interest over fairness. Hypocritical punishment remained unaffected by the manipulation of the contents of deliberation while the punishment bias was increased in the self-interest-deliberation condition compared to the fairness-deliberation condition. These findings validate that the punishment-enhancing effect of deliberating on fairness versus self-interest was specific to moral punishment.

## General discussion

Moral punishment of defection is essential for maintaining large-scale cooperation. People frequently accept costs to morally punish defection even if this does not yield any personal benefits for the punisher. The question prevails whether this individually irrational yet socially valuable behavior occurs intuitively, as predicted by an intuitive-morality account of punishment^10^, or whether it actually requires time and deliberation to overcome selfish incentive-driven impulses, as assumed by a deliberate-morality account of punishment^7^. Evidence on that matter is mixed and mainly stems from the Ultimatum Game which does not enable to clearly differentiate between participants’ inclination to cooperate and their inclination to punish^55^. In the present set of experiments, we relied on a one-shot simultaneous Prisoner’s Dilemma game with a punishment option. The cooperation-and-punishment model^5,56–58^ was applied to separately measure cooperation, moral punishment, hypocritical punishment and antisocial punishment as well as the punishment bias.

With respect to our initial question of whether the deliberate-morality account or the intuitive-morality account better explains how people apply punishment in social dilemmas, the conclusion is that the present results are more in line with the deliberate-morality account than with the intuitive-morality account of punishment. In Experiment 1, making punishment decisions with time pressure led to a decrease in moral punishment relative to a condition without time pressure, indicating that intuition tends to decrease moral behavior. These findings confirm and extend previous findings showing strikingly parallel effects to those of a cognitive-load manipulation^5^. Taken together, these findings suggest that the lack of cognitive resources decreases moral punishment, regardless of whether this lack of cognitive resources is caused by cognitive load or time pressure. In Experiment 2, punishment decisions were delayed by pauses in which participants were encouraged to deliberate their punishment decision. Deliberation led to an increase in moral punishment relative to a condition without deliberation. These findings support the deliberate-morality account of punishment by demonstrating that deliberation can facilitate the moral use of punishment. On the face of it, there is some plausibility to the idea that moral punishment requires deliberation. Punishment can be seen as a second-order social dilemma because participants may be torn between shying away from the costs of punishment which run against their self-interest and the moral motive of punishing unfair behaviors^90–92^. Deliberation may be needed to resolve these conflicting goals in favor of moral principles. Giving participants time to deliberate their decision may help to adhere to moral principles by suppressing their selfish impulses^51^. This is in line with neuro-imaging studies showing that punishment decisions in social dilemma games are accompanied by increased activation in brain areas associated with cognitive control^53,54^. It is also in line with research on moral behaviors more generally. For instance, there is evidence suggesting that people’s tendency to lie is increased when cognitive resources are limited, as telling the truth has been found to require self-control when lying serves immediate self-interests^30,31^. However, it is inconsistent with other findings showing evidence in the opposite direction^28,29^. Parallel to this, the present findings preserve the existing ambiguity regarding the intuitive versus deliberative nature of cooperation decisions^24^, with Experiment 1 showing no effect of time pressure on cooperation and Experiment 2 showing less cooperation with deliberation in comparison to the condition without deliberation.

Based on inconsistent findings regarding the effects of deliberation on moral decision making, Declerck and Boone^52^ have proposed a process-based account according to which the effect of deliberation on moral behaviors, such as cooperation and moral punishment, depends on contextual factors^8^. Several studies support this account^37,46,51,84,85^. The results of Experiment 3 extend these previous findings by demonstrating that explicit instructions on what to deliberate affect moral punishment: When participants deliberated fairness instead of self-interest, moral punishment increased.

Taken together, the findings of the present experiments thus suggest that, without manipulating the content of deliberation, unguided deliberation leads participants to use punishment in a moral way. However, the content of deliberation determines the effect of deliberation on moral punishment. Depending on the context in which the decision to punish has to be made, the content of deliberation may deviate from the moral default. Viewing punishment through the lens of the process-based account^52^ might be slightly more complex than postulating a strict dichotomy between intuitive and deliberate ways of arriving at a punishment decision, but adopting such a more nuanced approach offers great potential to integrate conflicting findings on the effects of intuition and deliberation on moral cognition^52,76^.

We conclude from the present findings that future research should focus on how the effects of intuition and deliberation on moral punishment are modulated by contextual factors. As an example of a potential avenue for future research, it seems possible to postulate that the effects of intuition on punishment should be context-dependent, just as the effects of deliberation on punishment observed in the present Experiment 3. That is, if heuristic cues favor moral or antisocial interpretations of situations, intuitions should favor or discourage punishment accordingly. Supporting this speculation, evidence suggests that the effect of intuition on punishment is modulated by contextual factors such as group membership^93,94^.

A limitation of the present study is that, in the present series of experiments, the social context of the interaction was emphasized, for instance, by presenting facial photographs of the interaction partners to take into account that everyday interactions often contain social cues. Given sufficient time for deliberation, this may have stimulated reflection on the social aspects of the interaction. This approach contrasts with the usual approach in Experimental Economics in which, even though social information is frequently manipulated, the games themselves are typically described in neutral terms. Another difference to the usual economic approach towards studying social interactions is that the participants interacted with simulated interaction partners, a standard practice in psychological research^63,95–97^. The experimental manipulation guarantees control over the partner’s behavior which is considered an extraneous factor in Experimental Psychology where the aim is to draw inferences about the cognitions underlying the individual’s behavior. It seems remarkable that participants in the present study punished their partners at a rate that is typical for studies involving human interaction partners even though this implied accepting real costs^21,98^. What is more, the fact that deliberation increased the probability of moral punishment suggests that the present paradigm taps into mechanisms of social interaction. Nevertheless, it has to be counted among the limitations of the present paradigm that participants interacted with programmed partners, particularly from the perspective of Experimental Economics where the primary focus lies on studying how incentive structures affect interactions in dyads or larger groups. It is therefore an interesting avenue of future research to test whether the present conclusions generalize to settings in which participants interact in human dyads or groups. It also seems important to note that the present set of experiments focused on one-shot interactions, that is, social situations during which participants interacted with each partner only once. More specifically, participants interacted with each of 20 different partners only once. One-shot interactions may be considered particularly relevant in that people in everyday life often invest own resources to punish strangers with whom they have interacted only once, which is an interesting phenomenon that requires explanation^58^. Even though one-shot interactions are generally assumed to be less influenced by factors such as reputation building or social learning, the repeated engagement of participants in the present Prisoner’s Dilemma game with different partners implies that an influence of these factors cannot be entirely ruled out. As a consequence, participants’ inclination to morally punish might have changed as participants progressed from the first interaction all the way to the final interaction. To examine whether this was the case, we tested whether a model would fit the data in which the moral-punishment parameter was set to be equal across the entire sequence of 20 interactions, separately for each of the two groups in Experiments 1, 2 and 3 (generating a total of 38 degrees of freedom for this statistical test in each experiment, 19 for the equality restriction of the moral-punishment parameter across the 20 interactions for each of the two groups). This equality restriction was compatible with the data in Experiment 1, Δ*G*^2^(38) = 48.86, *p* = 0.111, in Experiment 2, Δ*G*^2^(38) = 24.13, *p* = 0.960 and in Experiment 3, Δ*G*^2^(38) = 31.40, *p* = 0.767, leading to the conclusion that the parameter representing moral punishment did not change as participants progressed from the first interaction to the final interaction. This, in turn, suggests that in the present Prisoner’s Dilemma game the possible influence of factors such as reputation building or social learning on moral punishment is limited.

Another point worth discussing is that participants punished the partners they previously interacted with. As they directly suffered from their partners’ defection, the processes reflected in the moral-punishment parameter might go beyond motives that can, in a strict sense, be considered truly moral. For example, participants might punish to retaliate against the partners who have caused them harm^99^. It seems interesting to replicate the present findings in a paradigm that minimizes such self-interested motivations, for instance in a third-party-punishment paradigm in which the punishing party is not directly affected by the partner’s defection^100^.

## Conclusion

Do people punish defection intuitively or does this individually irrational, yet socially valuable, behavior rely on deliberation? Here we have found that time pressure decreased moral punishment (Experiment 1), which is parallel to what has been previously reported for a cognitive-load manipulation^5^, whereas deliberation during a time delay in which participants reflect on their punishment decision increased moral punishment (Experiment 2). Furthermore, we have demonstrated that moral punishment is affected by the specific content of deliberation (Experiment 3). When participants deliberate fairness, they are more likely to rely on moral punishment than when they deliberate self-interest. The results of Experiments 1 and 2 per se are more in line with the idea that moral punishment is applied in a deliberate rather than in an intuitive way. However, those results are also compatible with a more nuanced account supported by the results of Experiment 3 in which the effect of deliberation on moral punishment was modulated by contextual factors. Moving away from the strict dichotomy of classifying moral behaviors as either strictly intuitive or strictly deliberate has great potential in deepening our understanding of the effects on deliberation on punishment and offers a promising avenue for incorporating seemingly inconsistent findings by focusing on the cognitive processes underlying the observable behavior.

## Data Availability

We provide the data used in our analyses via the Open Science Framework. The study was not preregistered. The data are publicly available at https://osf.io/a23gy/.
